# Emerging Mechanisms of Growth and Patterning Regulation by Dachsous and Fat Protocadherins

**DOI:** 10.3389/fcell.2022.842593

**Published:** 2022-03-16

**Authors:** Artem Gridnev, Jyoti R. Misra

**Affiliations:** Department of Biological Sciences, University of Texas at Dallas, Richardson, TX, United States

**Keywords:** growth, morphogenesis, dachsous, fat-signaling, hippo signaling

## Abstract

Dachsous (Ds) and Fat are evolutionarily conserved cell adhesion molecules that play a critical role in development of multiple organ systems, where they coordinate tissue growth and morphogenesis. Much of our understanding of Ds-Fat signaling pathway comes from studies in *Drosophila*, where they initiate a signaling pathway that regulate growth by influencing Hippo signaling and morphogenesis by regulating Planar Cell Polarity (PCP). In this review, we discuss recent advances in our understanding of the mechanisms by which Ds-Fat signaling pathway regulates these critical developmental processes. Further, we discuss the progress in our understanding about how they function in mammals.

## 1 Introduction

Fat and Ds were originally discovered in *Drosophila*, based on lethality in the mutants and were subsequently shown to cause the dramatic overgrowth of the imaginal discs, the larval precursor for adult organs (Bryant et al., 1988; Clark et al., 1995). Later, they were shown to regulate orientation of wing hairs and ommatidia, the photoreceptor units of compound eye in *Drosophila*, by modulating PCP (Rawls et al., 2002; Strutt and Strutt, 2002; Yang et al., 2002). Subsequently, they were found to be conserved in mammals. These cell adhesion molecules are now known to play a critical role in coordinating growth and morphogenesis in developing organs from *Drosophila* to humans.

Molecularly, Fat and Ds are large single-pass transmembrane proteins with a large number of cadherin repeats in the extracellular domain (ECD) and a relatively small intracellular domain (ICD) (Mahoney et al., 1991; Tanoue and Takeichi, 2005). Unlike classical cadherins, their ICDs lack β-catenin binding sites. Mammals contain 4 Fat homologs (FAT1-4), out which FAT4 is closest to *Drosophila* Fat. Similarly, mammals have 2 Ds homologs (DCHS1 and DCHS2). These cell adhesion molecules interact in a heterophilic manner to initiate bidirectional signaling (hereafter Fat signaling), mediated by their cytoplasmic domains to regulate a number of critical developmental processes including growth, tissue patterning, convergent extension and directed cell migration. Mutations in FAT4 and DCHS1 are associated with many cancers and multi system developmental defects such as Van Maldergem syndrome and Hennekam syndrome, characterized by craniofacial anomalies, intellectual dysfunction, digital contractures, hypoplastic kidneys, sternal and auditory defects (Cappello et al., 2013; Alders et al., 2014; Hou et al., 2016; Ma et al., 2016; Pilehchian Langroudi et al., 2017).

**FIGURE 1:**
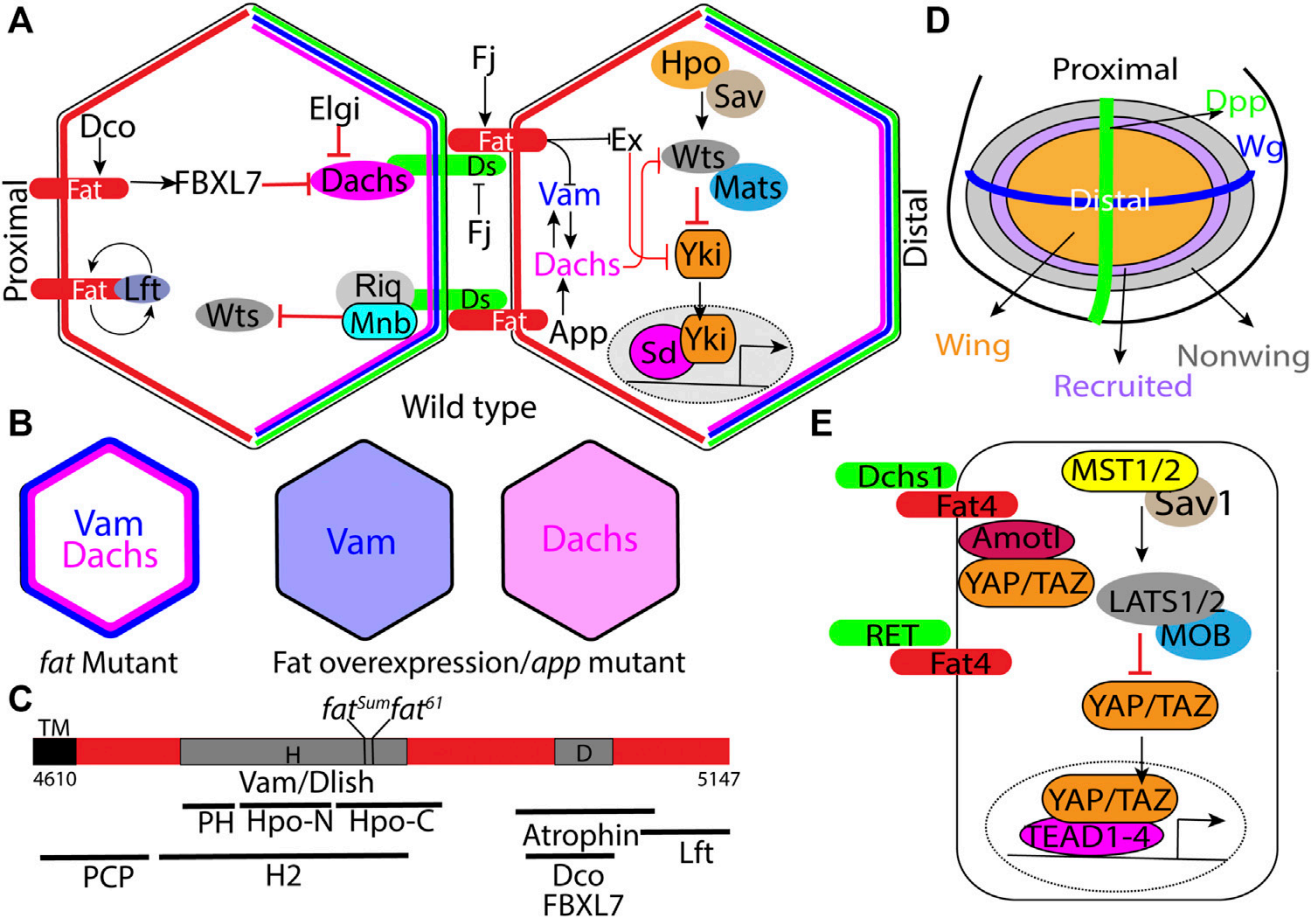
Regulation of Hippo signaling and tissue growth by Fat signaling. **(A)** Schematic of the minimal Ds-Fat signaling and the Hippo pathway showing asymmetric localization of Fat (proximally), Ds, Dachs and Vam (distally) in the apical cortex. **(B)** Schematic depicting loss of Dachs and Vam polarity in *fat* mutants, and displacement of Dachs and Vam from the membrane to the cytoplasm by Fat overexpression or in *app* mutants. **(C)** Schematic showing the H (aa 4,733–4,900) and D (aa 4,975–4,993) regions of the Fat intracellular domain, locations of point mutations within the H region, and deletions within the H region that impair Hippo activity, including HM (4,834–4,899), PH (4,733–4,774), Hpo-N (4,775–4,836), Hpo-C (4,839–4,920), and H2 (4,719–4,900) (Matakatsu and Blair, 2012; Pan et al., 2013; Zhao et al., 2013; Bossuyt et al., 2014). **(D)** Schematic depicting the *Drosophila* larval wing disc with Wg and Dpp expression domains along the dorsoventral and anterior-posterior boundary respectively and progressive enlargement of the wing pouch by recruitment of non-wing cells at the periphery by a feed-forward mechanism mediated by Fat signaling (see description in main text). Arrows and block arrows indicate positive and negative regulation respectively. **(E)** Schematic showing the simplified mammalian Hippo signaling pathway and regulation of RET and Yap/Taz (through Amotl1) by Fat4. Hpo: Hippo; Sav: Salvador; Wts: Warts; Mats: MOB as tumor suppressor; Yki:Yorkie; Sd: Scalloped; Ds: Dachsous; Fj: Four jointed; Dco: Discs overgrown; Lft:Low fat; Vam: Vamana/Dlish; Ex: Expanded; App: Approximated; Elgi: Early girl; Riq: Riquiqui; and Mnb: Minibrain.

Fat signaling regulates tissue growth by activating the Hippo signaling pathway, which consists of a core kinase module, consisting of Hippo (Hpo), Warts (Wts) and their cofactors Salvador (Sav) and MOB as tumor suppressor (Mats) (Kaishima et al., 2016) respectively, that function to regulate the transcriptional coactivator, Yorkie (Yki) (Misra and Irvine, 2018) (Figure 1A). Hpo phosphorylates and activates Wts, which in turn phosphorylates Yki. Phosphorylated Yki is sequestered in the cytoplasm, while unpophosphorylated Yki translocates into the nucleus and associates with the transcription factor Scalloped (Sd) to regulate the expression of genes that promote cell proliferation and inhibit apoptosis. This results in tissue overgrowth. This pathway is conserved in mammals and plays a central role in organ size control. In mammals, the Hpo homologs MST1/2 heterodimerize with the Sav ortholog SAV1 and phosphorylate Wts ortholog LATS1/2 and its adapter MOB1 (Mats ortholog) (Figure 1E). Phosphorylated LATS1/2 in turn, phosphorylate and sequester paralogous Yki orthologs, Yes Associated Protein (YAP) and Transcriptional activator with PDZ-binding motif (TAZ) in the cytoplasm. Unphosphorylated YAP/TAZ translocate into the nucleus, where they regulate gene expression by associating with Transcriptional Enhancer Associated Domain1-4 (TEAD1-4) transcription factors. The Hippo pathway integrates a diverse array of upstream biochemical, mechanical and architectural signals such as, cell-cell adhesion, cell polarity, cell geometry, hormones, nutrient status and cellular stress. In *Drosophila* Ds-Fat mediated cell-cell adhesion is a key upstream regulator of Hippo signaling. However, in mammals Dchs1/Fat4 influences this pathway only in specific tissues. Further, the mechanism by which they influence Hippo signaling seems to have evolutionarily diverged (Bossuyt et al., 2014).

**FIGURE 2:**
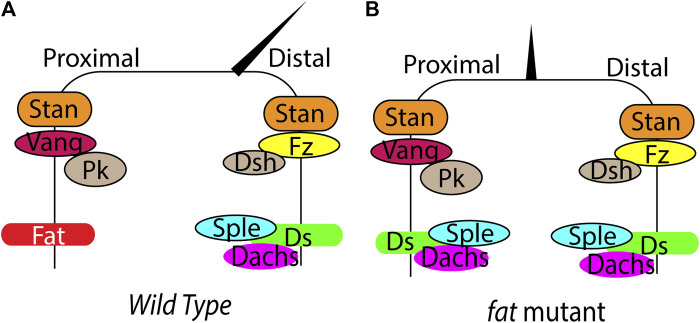
Regulation of PCP by Fat signaling in *Drosophila*. **(A) (B)** Schematic showing planar polarized localization of the core PCP components, Fat, Ds and hair orientation in wild type and *fat* mutant wings. In *fat* mutants, loss of Ds and Dachs polarity leads to loss of Sple asymmetry, causing loss of hair polarity.

Fat signaling also regulates tissue morphogenesis by influencing PCP, which refers to tissue wide coordinate polarization of cellular features in an organ, in the plane of the tissue (Strutt and Strutt, 2021). For example, the hairs in the *Drosophila* adult wings and abdomen, and the hair follicles in mammalian skin uniformly point to one direction. Similarly, the stereocilia in the cochlear hair cells in the mammalian inner ear are also uniformly organized, and disorganization of this leads to deafness. PCP is primarily regulated by a conserved signaling network mediated by the core pathway of PCP proteins, which consists of the transmembrane proteins Starry night (Stan) (also known as Flamingo), Frizzled and Vangogh (Vang) (also known as Strabismus), and the cytosolic proteins Prickle (Pk), Disheveled (Dsh) and Diego (Dgo) (Goodrich and Strutt, 2011). These proteins localize in asymmetric complexes in a planar polarized manner to regulate PCP (Figure 2A). Fat signaling can regulate tissue patterning by influencing the polarization of these core proteins.

Here we will first describe recent advances in our understanding of Fat signaling in *Drosophila*, where the pathway is most extensively studied. Then we will summarize our understandings of how these protocadherins function in mammals.

## 2 FAT Signaling in *Drosophila*

### 2.1 Pathway Components

#### 2.1.1 Fat and Ds

*Drosophila* Fat is a very large (560 KD) transmembrane protein with 34 cadherin repeats, 4 EGF like repeats and 2 lamin-G domains in the ECD. This is followed by a transmembrane domain and relatively small ICD, which does not have any identifiable domains. However, specific regions in the ICD have been identified that play important role in growth or PCP regulation (Figure 1C). The mature protein exists in a form cleaved N-terminal to the transmembrane domain (Feng and Irvine, 2009; Sopko et al., 2009). Ds is also a very large (379 KDa) transmembrane domain with 27 cadherin repeats in the ECD, followed by a transmembrane region and a small ICD. Cellular aggregation experiments using S2 cells expressing these proteins revealed that they mediate cell-cell adhesion by interacting in a heterophilic manner (Matakatsu and Blair, 2004). Subsequent rescue experiments in *fat* and *ds* mutants, which exhibit overgrowth of the imaginal discs and lethality, revealed that, these phenotypes in *fat* mutants can be rescued by expressing just the Fat-ICD. Similarly, expression of just the Ds-ECD can rescue overgrowth phenotypes in *ds* mutants. These experiments showed that Fat functions as the receptor and Ds functions as the ligand (Matakatsu and Blair, 2004; Matakatsu and Blair, 2006; Rogulja et al., 2008; Willecke et al., 2008). However, Ds is also thought to function as a receptor in specific circumstances (Zecca and Struhl, 2010).

Much of our understanding of the molecular mechanism by which this pathway regulates growth and morphogenesis comes from studies in the *Drosophila* wing imaginal disc, the primordium that gives rise to the adult wing. In the *Drosophila* wing disc epithelial cells, Fat and Ds localize to the subapical plasma membrane in a planar polarized manner, where Fat preferentially localizes to the proximal side and Ds localizes to the distal side (Ambegaonkar et al., 2012; Brittle et al., 2012). This facilitates heterophilic interaction between Ds and Fat across cell-cell junctions. Ds-Fat interaction is also modulated by phosphorylation of their ECDs by the Golgi-resident kinase Four jointed (Fj), where Fat phosphorylation promotes its interaction with Ds, while Ds phosphorylation is known to inhibit its interaction with Fat (Ishikawa et al., 2008; Brittle et al., 2010; Simon et al., 2010; Hale et al., 2015).

One of the unique feature of this Fat signaling pathway is that the signaling activity is dependent on protein expression gradients. Under the influence of the Vestigial (Vg) transcription factor, Ds is expressed in a steep decreasing gradient from the periphery to the center of the wing pouch. On the other hand, Vg promotes Fj expression in an opposite decreasing gradient from the center to the periphery of the presumptive wing primordium (Cho and Irvine, 2004). Computational modeling revealed that the graded expression of Ds and Fj results in planar polarization of Fat and Ds along the proximodistal axis (Hale et al., 2015). While Ds is expressed in a gradient, Fat is expressed almost uniformly. This results in a gradient of Fat activity, and the differential Fat signaling between adjacent cells is known to regulate cell proliferation by activating Yki. Consistently, creating sharp differences in Fat signaling by expressing Ds in clones, activates Yki in the clone boundary (Rogulja et al., 2008; Willecke et al., 2008). Conversely, flattening the gradient by uniformly expressing Fat, Ds or Fj inhibits growth (Matakatsu and Blair, 2006). While the gradient of Fat activity is responsible for sustaining the growth and proliferation of the wing cells in the pouch region, it has also been proposed that at the periphery of the wing pouch, Fat and Ds also contribute to wing growth by propagating a feedforward mechanism to recruit non-wing cells into the wing pouch (Figure 1D) (Zecca and Struhl, 2010). This mechanism relies on steep borders of Ds and Fj expression at the edge of the developing wing.

#### 2.1.2 Dachs

Dachs is one the key downstream effector of the Fat, and was isolated based genetic epistasis experiments where *dachs* mutants suppressed the lethality and overgrowth phenotypes of *fat* mutants (Cho et al., 2006; Mao et al., 2006). Molecularly, it encodes an atypical myosin with no ATPase activity that can bind to F-actin (Cao et al., 2014). Cell biological studies revealed that in the developing wing disc epithelial cells, it localizes to the subapical plasma membrane in a planar polarized manner, with enrichment on the distal side (Figure 1A). (Mao et al., 2006). In *fat* mutants however, Dachs levels increase and it localizes to the entire perimeter of the cells (Figure 1B). Further, forced localization of Dachs to the entire perimeter by fusing it to Zyxin can induce similar overgrowth as *fat* mutants (Pan et al., 2013). Conversely, overexpression of Fat or Fat-ICD displaces membrane-bound Dachs into the cytoplasm (Figure 1B). Thus, membrane localization is critical to Dachs function and its polarity plays a crucial role in regulation of growth *via* the Hippo signaling pathway. Fat regulates growth by regulating Dachs levels, membrane localization and polarity. Dachs levels and polarity is also regulated by two E3 ubiquitin ligases, Early girl (elgi) and FBXL7 respectively (Bosch et al., 2014; Rodrigues-Campos and Thompson, 2014; Misra and Irvine, 2019). FBXL7 mutants exhibit a milder increase in Dachs levels that lose the polarity, which induces overgrowth. On the other hand, *elgi* mutants display dramatic increase in Dachs levels that shows normal polarity and mild overgrowth.

#### 2.1.3 Approximated

A hallmark feature of mutations in Fat signaling pathway is that they display reduced spacing between two vertical thickenings in the wing, referred to as crossveins. Approximated (App) was isolated based on the reduced crossvein spacing phenotypes in the adult wings of the mutant animals (Matakatsu and Blair, 2008). App encodes a DHHC palmitoyl transferase and at the cellular level, *app* mutants exhibit reduced membrane localization of Dachs, indicating that it is required for proper localization of Dachs (Figure 1B). However, App does not palmitoylate Dachs directly. App has been also reported to palmitoylate Vamana (Zhang et al., 2016) and juxta membrane region of Fat and regulate their membrane localization (Matakatsu et al., 2017).

#### 2.1.4 Vamana/Dachs Ligand With SH3 Domains

Vamana (Vam) [also known as Dachs ligand with SH3 domains (Dlish)] was isolated by two groups independently (Misra and Irvine, 2016; Zhang et al., 2016). Misra and Irvine (2016) discovered Vam, based on a reduced crossvein spacing of the adults wings from a stock containing a transposable element inserted into the Vam locus. Zhang et al. (2016) isolated Dlish as an interactor of the Dachs C-terminal region in a yeast two hybrid screen. Vam mutants exhibit undergrowth and can suppress the overgrowth phenotype of Fat mutants. Vam encodes an adapter protein with 3 SH3 domains and physically interacts with Dachs, engaging the second SH3 domain. In absence of Vam, Dachs fails to localize to the plasma membrane. Conversely, Vam also fails to localize to the membrane in absence of Dachs, suggesting that they reciprocally regulate each other. Consistent with this, Vam localizes to the distal side of apical plasma membrane, where it colocalizes with both Dachs and Ds (Figure 1A). Interestingly, Fat regulates Vam level and polarity in the same manner as it regulates Dachs. In absence of Fat, Vam levels also increases and it localizes to the entire perimeter of the cells (Figure 1B). Conversely, overexpression of Fat or Fat-ICD displaces Dachs from the membrane to the cytoplasm (Figure 1B). Although it was previously shown that Fat negatively regulates Dachs, there was no evidence of direct physical interaction between Fat and Dachs. Vam provided the physical link between Dachs and, both Fat and Ds. The first and third SH3 domains of Dachs interact with the cytoplasmic domains of Fat and Ds. More importantly, these SH3 domains interact with the Hippo regulatory (H) region of the Fat-ICD (Figure 1C). Vam was also subsequently shown to regulate expanded stability by recruiting the E3 ligase Slimb (Wang et al., 2019). Thus, in absence of Vam, expanded levels are significantly increased. However, overexpression of Vam has no apparent effect on Expanded (Ex) levels. Thus, it remains unclear how a modest increase in Vam levels in *fat* mutants can significantly destabilize Ex levels.

#### 2.1.5 Expanded

Expanded (Ex) is a FERM domain protein that functions as a crucial negative feedback regulator in the Hippo signaling pathway. Ex is transcriptionally induced by activated Yki and it localizes to the apical plasma membrane by interacting with the cytoplasmic domain of the cell adhesion protein Crumbs (Chen et al., 2010; Ling et al., 2010; Robinson et al., 2010). It directly binds to Yki and sequesters it in the membrane (Badouel et al., 2009; Oh et al., 2009). Recent studies revealed that as a negative feedback loop, Wts gets activated at the plasma membrane and Ex functions as a scaffold that interacts with Hippo, Wts and Yki. Thus, it plays a critical role in promoting the Hpo-Wts kinase cascade to restrict Yki activity (Sun et al., 2015). Therefore, in absence of Ex, Yki is presumably activated in an uncontrolled manner. Ex protein stability is also regulated by Crumbs (Chen et al., 2010; Ribeiro et al., 2014; Fulford et al., 2019). In absence of Crumbs, Ex fails to localize to the plasma membrane. Conversely, higher levels of crumbs induces Ex ubiquitination and subsequent degradation by recruiting the E3 ligase Slimb, through the cytoplasmic domain. Fat mutants display reduced apical Ex protein levels, despite increased *ex* transcription by activated Yki and this is thought to be the key mechanism by which loss of Fat induces overgrowth (Bennett and Harvey, 2006; Silva et al., 2006; Willecke et al., 2006; Feng and Irvine, 2007). However, another school of thought is that Fat and Ex function in parallel to regulate growth through Hippo signaling (Feng and Irvine, 2007). Concomitant loss of Dachs and Fat restores Ex levels in *fat* mutants, presumably due to absence of Vam, which also promotes Ex ubiquitination and degradation through Slimb. It remains unclear how exactly Fat regulates Ex protein stability.

#### 2.1.6 Discs Overgrown

Discs overgrown (Dco), as the name suggests, was isolated based on the overgrowth of the wing discs in animals carrying a neomorphic gain-of-function mutant allele, *Dco3*. *Dco3* mutants also display higher levels of Dachs. Dco encodes Casein Kinase-1ε and phosphorylates Fat-ICD in the D region (Figure 1C), which then recruits FBXL7 that promotes Dachs degradation and restricts it to the distal side (Bosch et al., 2014). Thus, in *Dco^3^* mutants, loss of FBXL7 function interferes with Dachs polarity and leads to overgrowth.

#### 2.1.7 Low Fat

Low fat (Lft) was reported as an interactor of Fat and Ds ICDs in a yeast two-hybrid screen. Subsequent genetic analysis revealed that the *lft* mutants exhibit reduced crossvein spacing (Mao et al., 2009). At the cellular level, loss of Lft led to a decrease in Fat and Ds levels. Conversely, overexpression of Lft promoted Fat and Ds membrane recruitment. Lft is evolutionarily conserved and the human homologs LIX1 and LIX1L can suppress the *Drosophila* Lft mutant phenotypes. However, how exactly, Lft regulates Fat or Ds remains unknown.

#### 2.1.8 Atrophin

Atrophin (Atro) (also known as Grunge) is a downstream effector of Fat in PCP regulation in the eye (Fanto et al., 2003). Atro is a transcriptional repressor and physically associates with the Fat-ICD. In absence of Fat, it translocates into the nucleus and regulates gene expression. However, the transcriptional target of Atro that contributes to PCP regulation remains to be identified.

### 2.2 Fat and Hippo Signaling

Fat signaling primarily restricts tissue growth by activating Hippo signaling. Genetic epistasis experiments revealed that Fat regulates Hippo pathway at the level of Wts (Cho et al., 2006). However, the exact mechanism by which it regulates Wts remains unclear. One school of thought is that Fat regulates Wts stability. It was shown that in *fat* mutants as well as cells expressing Dachs fused to Zyxin, Wts is destabilized (Cho et al., 2006; Rauskolb et al., 2011). However, how exactly Fat regulates Wts stability remains unknown. Another model proposes that Fat regulates Wts activity (Vrabioiu and Struhl, 2015). Using Fluorescence Resonance Energy Transfer (FRET) based Wts constructs, the authors showed that Wts remains in a closed inactive conformation and open active conformation. Mats induces active open conformation and Dachs reverses or inhibits this switch. Thus, in absence of Fat, where there is increased amount of Dachs that localizes to the entire circumference of the cells, it would be expected to promote close inactive conformation of Wts. However, in *elgi* mutants, where Dachs levels are very high but still polarized, Wts can still remain in active conformation, to restrict growth (Misra and Irvine, 2019). However, active open conformation of Wts does not show any apparent planar polarization. While it is possible that interaction between Dachs and Wts could be transient, it remains an open possibility that Dachs could regulate Wts through a different mechanism. Fat is also known to regulate Hippo signaling by affecting expanded stability. However, the exact mechanism by which it regulates Ex remains unknown. It is important to note that while Fat can regulate Wts through Dachs in wing discs, Dachs is not expressed in all tissues. For example, Fat regulates Hippo signaling in the eye in a Dachs independent manner. Thus, Fat regulates Hippo pathway activity in different tissues through distinct mechanisms.

To gain insight into the mechanism by which Fat-ICD regulates Hippo signaling and PCP, several groups have made deletions in conserved blocks of amino acids and examined their function (Matakatsu and Blair, 2012; Pan et al., 2013; Zhao et al., 2013). These studies revealed that there are distinct regions in Fat-ICD that regulate growth and PCP (Figure 1C). For example, these studies identified two regions that contribute toward growth regulation. One region encompassing amino acids 4,975 to 4,993, referred to as the D region makes a moderate contribution to growth, as mutants lacking this region are viable and the wings are only 30% overgrown. The D region is necessary for recruitment of FBXL7, which regulates Dachs levels and polarity. Another region referred to as H/HM/H2 region plays a more critical role, as flies lacking this region fail to survive. Further, *fat* ^*sum*^ and *fat* ^*61*^ mutants that harbor mutations in this region exhibit same phenotype as *fat* null mutants (Bosch et al., 2014). Interestingly, this region binds to Vam/Dlish to regulate Dachs.

### 2.3 Fat and Mitochondria

Fat also regulates metabolism, which influences growth, Hippo signaling and PCP. Fat cytoplasmic domain contains multiple mitochondrial targeting signal and is cleaved to produce a fragment that translocates into the mitochondria and stabilizes complex I (Sing et al., 2014). *fat* mutants display reduced amount of Complex I and switch to glycolytic metabolism. Interestingly, disrupting components of complex I also causes PCP defects in the eye suggesting that mitochondrial signals may influence PCP.

### 2.4 Ds and Hippo Signaling

*ds* mutants also display overgrowth phenotype by activating Yki, although the phenotype is not as severe as in *fat* mutants. This is presumably because residual amount of Fat is still present in *ds* mutants that retains significant amount of ligand independent activity. Expression of Ds-ICD can also activate Yki and this could be partly due to recruiting more Dachs through Vam, which then inhibits Wts activity. In addition, Ds-ICD promotes growth through a second mechanism by recruiting Riquiqui and Minibrain DYRK kinase to the membrane which phosphorylates and inhibits Wts activity (Figure 1A) (Degoutin et al., 2013).

### 2.5 Fat Signaling in Planar Cell Polarity

Fat and Ds regulate tissue patterning by influencing the core PCP signaling. Both Ds, Fat and the core PCP proteins localize in an asymmetric manner creating cellular anisotropies that regulates PCP (Figure 2A). Ds and Fat asymmetry is established by the opposing gradients of Ds and Fj. The differential expression of Ds and Fj in neighboring cells affects their binding properties and polarizes Ds-bound Fat and Fat-bound Ds to opposite sides. Development of robust Ds-Fat polarization from slight initial differences possibly requires one or more type of amplifications. However, the amplification mechanisms are currently remain unknown. Once established, the Ds-Fat polarity must be transduced to polarized cellular structures. Here also Dachs plays an important role. The gene *prickle* (*pk*) provides a connecting link between core PCP and Fat signaling. *pk* encodes Prickle and Spiny-legs (Sple) proteins and the relative ratio of the two isoforms regulate core PCP polarization relative to Ds-Fat (Ayukawa et al., 2014; Olofsson et al., 2014). Tissues with high level of Pk bias the plus end of microtubules towards low Ds side. In contrast, tissues with high Sple exhibit plus end of microtubules biased to high Ds side. This affects core PCP polarization through transcytosis of Dsh along the microtubules (Matis et al., 2014; Olofsson et al., 2014). Ds and Dachs physically interact with Sple and couple the core PCP with Fat-Ds. Changing the levels of Pk/Sple ratio regulates coupling and uncoupling between Ft/Ds and core modules (Figure 2B) (Ayukawa et al., 2014; Ambegaonkar and Irvine, 2015). However, it remains disputed whether Fat/Ds system instructs the core PCP pathway in *Drosophila* abdomen.

Fat and Ds also regulate PCP in a Dachs independent manner. The transcriptional corepressor Atrophin binds to the cytoplasmic domain of Fat and regulates PCP in the equatorial region of the eye (Fanto et al., 2003). However, the exact transcriptional target of Atrophin that influences PCP remains unknown. Fat signaling also regulates other forms of cell polarity such as oriented cell division, oriented cell tensions, and orientation of larval denticle belts independent of the core PCP system (Mao et al., 2011b; Donoughe and DiNardo, 2011; Lawlor et al., 2013).

### 2.6 Fat Signaling in Junctional Tension

Fat, Ds and Dachs also play an important role in polarization of adherens junction tension (Mao et al., 2011b). Laser ablation experiments revealed that *fat* mutant clones exhibit higher tension at the clone border abutting the wild type tissue but show less tension within the clones (Bosveld et al., 2016). Dachs is necessary for these effect on junctional tension, by directly accumulating at the clone border and indirectly by decreasing internal tension due to increase cell proliferation by inhibiting the Hippo pathway (Bosveld et al., 2012).

## 3 FAT Signaling in Mammals

In contrast to Fat signaling in *Drosophila*, Fat signaling in vertebrates has diverged through evolution. The key downstream components such as Dachs, Vam/Dlish and Ex are not conserved in mammals (Bossuyt et al., 2014). Although Fat4 ICD contains conserved blocks of amino acids, it fails to regulate Hippo and PCP in flies (Pan et al., 2013). Mutations in Fat4 and DCHS1 are associated with Van Maldergem and Hennekam syndrome and many cancers (Mansour et al., 2012; Cappello et al., 2013; Alders et al., 2014; van der Ven et al., 2017). Mouse knock outs of Fat4 and Dchs1 exhibit developmental defects in kidney, brain, lymphatic and skeletal systems (Saburi et al., 2008; Mao et al., 2011a; Saburi et al., 2012; Zakaria et al., 2014; Durst et al., 2015; Kuta et al., 2016; Crespo-Enriquez et al., 2019). In most cases, loss of Fat-4 leads to underproliferation and affects the number of neuronal, nephrogenic and chondrocyte progenitors, their polarity, and neural migrations. However, the underlying mechanisms are less well understood. In kidney, Fat4 binds to and modulates RET receptor tyrosine kinase signaling, so that Fat4 mutants exhibit excessive RET signaling, leading to abnormal ureteric budding (Figure 1E) (Zhang et al., 2019). In mouse brain and human cerebral organoids loss of Fat4 or DCHS1 impacts neuronal proliferation, differentiation and migration in a YAP/TAZ independent manner (Cappello et al., 2013; Klaus et al., 2019). In specific cases, Fat4 and DCHS1 induce cell proliferation in a YAP/TAZ dependent manner. For example, Fat4 regulates growth of cardiac tissue by sequestering YAP/TAZ by interacting with Angiomotin like-1 (Amotl1) (Figure 1E) (Ragni et al., 2017). Fat4 mutations are also associated with abnormal cortical development due to increased YAP activity and neuronal differentiation.

## 4 Concluding Remarks

Precise coordination of growth and morphogenesis during development is critical to formation of optimally functioning organs, and Fat signaling plays a central role in coordinating these processes. Although there has been significant progress in our understanding of this pathway both in *Drosophila* and mammals in the last few years, our knowledge of this pathway still remains rudimentary. It is not completely understood how Fat regulates Hippo pathway. Further, loss of Fat/Ds in *Drosophila* and Fat4/Dchs1 in mice results in altered aspect ratio of the organs. It remains a challenge to identify the mechanisms by which these protocadherins regulate organ shape. Further, it will be important to understand the molecular basis underlying the differences in signaling output in different tissues. Future studies in *Drosophila* and mammals will provide a unified mechanism by which Ds and Fat coordinate growth and morphogenesis in multiple organs.
